# Surfactant protein D: prognostic insights and emerging therapeutic roles in cancer

**DOI:** 10.3389/fimmu.2026.1870290

**Published:** 2026-07-09

**Authors:** Sharon Ajay Mathew, Sushama Rokade, K.G. Aghila Rani, Hrishikesh Pandit, Uday Kishore, Taruna Madan

**Affiliations:** 1 Department of Life Sciences, Somaiya School of Basic and Applied Sciences, Somaiya Vidyavihar University (SVU), Mumbai, India; 2 Department of Innate Immunity, Indian Council of Medical Research (ICMR)- National Institute for Research on Women’s Health, Mumbai, India; 3 Research Institute for Medical and Health Sciences, University of Sharjah, Sharjah, United Arab Emirates; 4 Laboratory of Molecular Immunology and the Immunology Center, National Heart, Lung and Blood Institute, National Institutes of Health, Bethesda, MD, United States; 5 Department of Veterinary Medicine (CAVM) & Zayed Centre for Health Sciences, United Arab Emirates University, Al Ain, United Arab Emirates; 6 Development Research, Indian Council of Medical Research, New Delhi, India

**Keywords:** biomarker, cancer, immune surveillance, oncogenic pathogens, prognosis, surfactant protein D

## Abstract

Surfactant protein D (SP-D), a C-type lectin found at the mucosal surfaces, plays crucial roles in innate immunity and homeostasis of the lung. In addition to the lungs, extra-pulmonary expression of SP-D has been reported in several tissues. Differential expression of SP-D in lung adenocarcinoma and metastases implicated its importance in diagnosis and prognosis. Serendipitous discovery of the direct anti-tumor effect of SP-D in human eosinophilic leukemia and breast cancer cells opened the field for exploring its therapeutic potential in various cancers and its contribution to immunomodulatory mechanisms. The current review discusses the role of SP-D as a prognostic marker in lung, breast, gastrointestinal, pancreatic, ovarian and prostate cancer. Additionally, levels of SP-D in serum and bronchoalveolar lavage enable diagnosis and assessment of the risk of developing lung cancer. The molecular interaction of SP-D with Epidermal Growth Factor Receptor and Glucose-Related Protein 78, induction of cell cycle arrest and activation of the intrinsic apoptosis pathways are some of the salient mechanisms contributing to the immune surveillance of tumor cells by SP-D. The review also discusses SP-D interactions with oncogenic pathogens and therapeutic potential of recombinant SP-D.

## 1 Introduction

**Figure 1:**
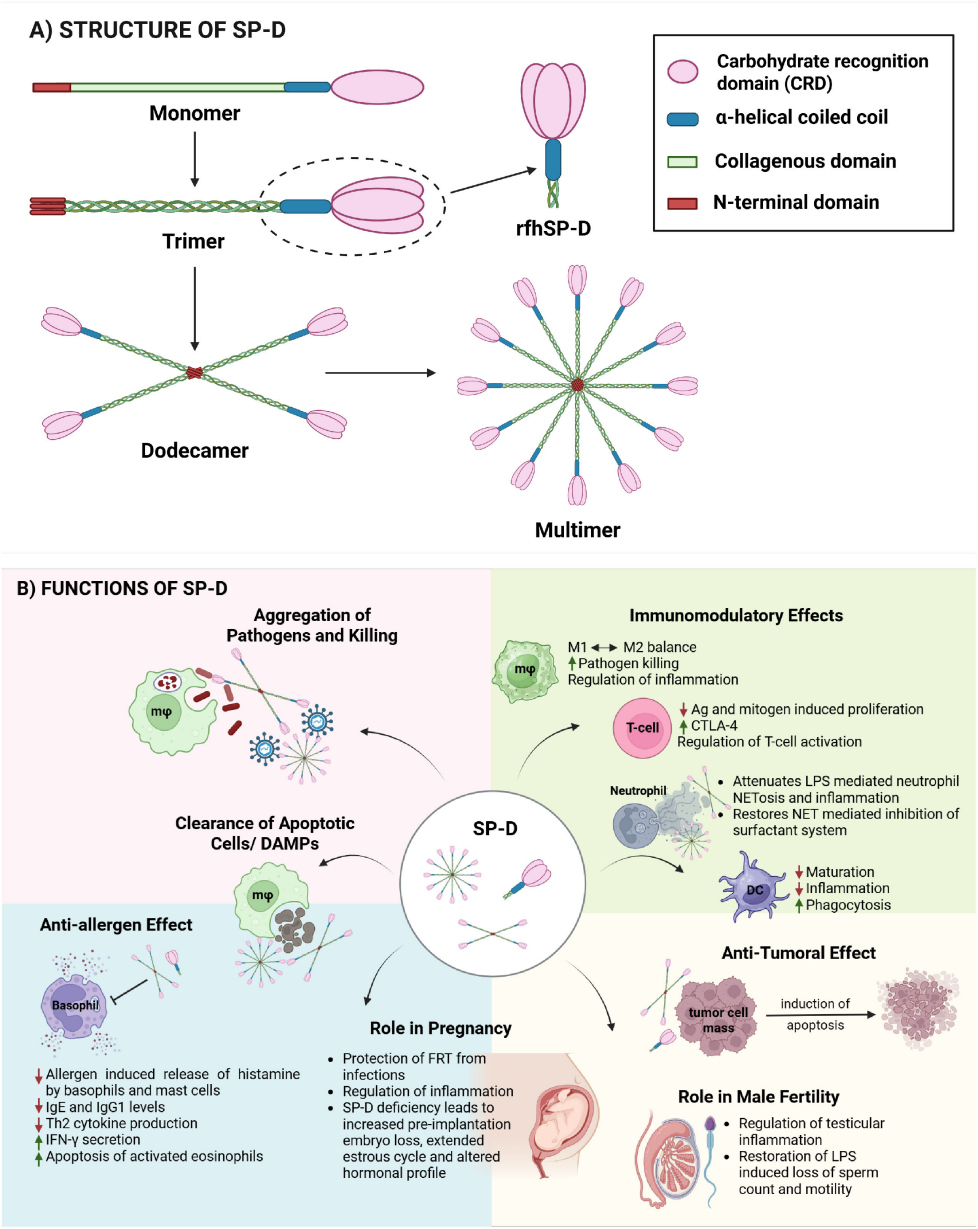
Structure and multifunctional roles of SP-D. **(A)** SP-D is composed of homotrimeric polypeptide chains, each containing an N-terminal cysteine-rich region, a collagen-like domain, an α-helical coiled-coil neck region, and a C-type lectin or carbohydrate recognition domain (CRD). These trimers further oligomerize via their N-terminal domains to form higher-order tetrameric structures. A recombinant human fragment of SP-D (rfhSP-D) consists of CRD, trimeric neck regions and a collagenous part containing eight Gly-X-Y repeats. **(B)** Functionally, SP-D plays a central role in innate immunity by facilitating the opsonization and clearance of apoptotic cells, exerting antimicrobial activity via direct pathogen aggregation and lysis, and preserving mucosal barrier integrity. Additionally, SP-D exerts an anti-allergen effect by recognizing a range of allergens, directly competing with specific IgE antibodies and blocking the release of histamine by mast cells and basophils. It can inhibit Th2 cytokine production by allergen activated T cells and induce apoptosis of activated eosinophils. It plays a critical role in regulating immune homeostasis by modulating the activity of macrophages (mØ), dendritic cells (DC), T-cells and neutrophils, thus preventing excessive inflammation that could lead to tissue damage. Furthermore, SP-D exerts anti-tumoral effects by inducing apoptosis of cancer cells. It also regulates LPS mediated inflammatory responses in the testis and rescues the inflammation induced decrease of sperm count and motility. In female reproductive tract, SP-D protects against pathogens and modulates inflammation during pregnancy. Deficiency of SP-D is linked to increased pre-implantation embryo loss, extended estrous cycles, and altered ovarian hormone profiles, suggesting that it is critical for fertility. *Image created in Biorender*. https://BioRender.com.

Surfactant protein D (SP-D) is a multifunctional soluble glycoprotein, belonging to the family of collagen containing C-type lectins, called Collectins. It is known to be primarily involved in maintaining pulmonary homeostasis and mediating innate immune responses against pathogens (1). Structurally, SP-D consists of homotrimeric polypeptide chains, each composed of four major domains: an N-terminal cysteine-rich region, a triple helical collagen-like domain, an α-helical coiled-coil neck region, and a carbohydrate recognition domain (CRD) or C- type lectin domain. These homotrimeric subunits further oligomerize to form tetrameric structures linked *via* their N-terminal domain (2) (**Figure 1A**). In the lungs, SP-D is predominantly synthesized and secreted by type II alveolar cells and Clara cells (3). Studies during the last two decades have established its extrapulmonary existence in a variety of tissues indicating wider physiological significance of SP-D (4–6).

SP-D is known to regulate multiple biological processes including the clearance of pathogens and apoptotic cells, maintenance of the integrity of mucosal barrier, and regulation of inflammatory responses (2, 7). It can bind to a range of allergens, competing with specific IgE antibodies and reducing histamine release from basophils and mast cells (8). It can induce apoptosis of allergen activated eosinophils (9). Furthermore, SP-D plays a critical role in regulating immune homeostasis by modulating the activity of macrophages, dendritic cells, neutrophils and T cells, thus preventing excessive inflammation that could lead to tissue damage (10). Beyond its role in pathogen defense, SP-D also has a regulatory effect on cellular proliferation, apoptosis, and oxidative stress (11). Thus, SP-D functions as a crucial regulator of tissue homeostasis, particularly evident in inflammatory and fibrotic lung diseases such as chronic obstructive pulmonary disease (COPD) and idiopathic pulmonary fibrosis (IPF) (12). Additionally, the role of SP-D in fertility has also been explored. During pregnancy, SP-D protects the female reproductive tract and fetus against infections and regulates inflammation. The deficiency of SP-D in murine models has been associated with increased pre-implantation embryo loss, extended estrous cycles, and altered ovarian hormone profiles, suggesting its crucial role in fertility (13, 14). In the male reproductive tract, SP-D regulates LPS mediated inflammatory responses in the testis and restores the inflammation induced decrease of sperm count and motility (15) (**Figure 1B**).

Given its multifaceted roles in immune regulation and epithelial integrity, SP-D’s involvement in cancer biology has gained increasing attention. In the very first report (16), detection of surfactant protein-A (SP-A), surfactant protein-C (SP-C), or SP-D were shown by reverse transcriptase (RT)-PCR as tumor markers, serving as a valuable tool to detect metastatic pulmonary adenocarcinomas. Gene expression of SP-A and SP-C was shown to be restricted to metastatic pulmonary adenocarcinomas; SP-D transcripts were shown to be expressed in metastases of pulmonary large cell carcinomas, adenosquamous carcinoma, and extrapulmonary adenocarcinomas. Altered expression and activity of SP-D have been associated with various malignancies, including lung, breast, gastric, ovarian, prostate, pancreatic and colorectal cancers (9, 17–23). Mahajan et al. demonstrated that purified human SP-D and its recombinant fragment composed of 8 gly-x-y repeats, homotrimeric neck and CRD region (rfhSP-D) selectively reduced the survival of various cancerous cells such as AML14.3D10, Jurkat, Raji, MCF-7 and THP-1 cells, without affecting healthy peripheral blood mononuclear cells (PBMCs) (9). This provided the first direct evidence of the tumoricidal activity of SP-D. In this study, SP-D and rfhSP-D induced G2/M phase cell cycle arrest, and dose and time-dependent apoptosis in the AML14.3D10 eosinophilic leukemia cell line through the p53 pathway (9). Further, SP-D has been shown to downregulate the epidermal growth factor (EGF) signaling pathway through direct interaction with EGF receptor (EGFR), thus inhibiting cell proliferation, invasion, and metastasis in the A549 lung cancer cell line (18). The rfhSP-D treatment of the phytohemagglutinin (PHA) activated PBMCs, led to apoptosis in the activated but not in non-activated lymphocytes through up-regulation of cytotoxic T-lymphocyte associated antigen 4 (CTLA4) (25). In pancreatic cancer, rfhSP-D induced apoptosis *in vitro via* the Fas-mediated pathway (19). Additionally, rfhSP-D suppressed transforming growth factor β (TGF-β) expression in several pancreatic cancer cell lines and reduced their invasive capacity by inhibiting the epithelial-to-mesenchymal transition (EMT) (20).

Thakur et al. reported that rfhSP-D triggered cell death in prostate cancer explants and primary tumor cells derived from metastatic prostate cancer patients *via* p53 and pAkt pathways (22). Significantly decreased SP-D mRNA expression and increased proteolytic degradation of SP-D protein were demonstrated in both early and advanced stages of prostate cancer using transgenic adenocarcinoma of mouse prostate (TRAMP) model, driven by increased secretion of serine proteases and elastases in tumor microenvironment (TME) (24). These findings indicate that SP-D plays an anti-tumorigenic role in prostate cancer while the prostate tumor milieu adversely impacts SP-D by inhibiting its transcription and enhancing its proteolytic degradation. Conversely, in breast and ovarian cancers, elevated levels of SP-D correlated with a poor prognosis, suggesting that SP-D exerted tissue- or cell-specific effects that were contingent upon the TME (21). Deciphering the molecular mechanisms by which SP-D interacts with tumor cells and the immune system is critical for unraveling its diverse roles in tumorigenesis.

Current cancer immunotherapies predominantly focus on harnessing the adaptive immune system, particularly through immune checkpoint inhibitors targeting T cells [e.g., programmed cell death protein 1 (PD-1), programmed cell death ligand 1 (PD-L1), CTLA4] (26). While these therapies have revolutionized cancer treatment, they are limited by resistance to the treatment and inefficacy in a significant subset of patients. This underscores the need to explore the therapeutic potential of the innate immune system which provides a broader and often more immediate response against tumors. Molecules such as stimulator of interferon genes (*STING)* agonists, which activate type I interferon responses; NOD (nucleotide-binding oligomerization domain)-, LRR (Leucine-rich repeat)- and pyrin domain-containing protein 3 (*NLRP3) inflammasome* modulators, which reshape the TME to enhance immune infiltration; and toll like receptor (TLR) agonists, which activate the immune system are currently in preclinical and clinical trials showing promise in promoting anti-tumor immunity (27). SP-D reduces CD11c expression (25), a marker primarily found on macrophages and dendritic cells. CD11c is also present in Natural Killer (NK) cells and activated T cells (28). These immune cells collectively play a critical role in managing immune surveillance and anti-tumor responses. Thus, SP-D could also offer a promising avenue for cancer immunotherapy owing to its ability to modulate immune responses and directly inhibit tumorigenesis as well as tumor progression.

This review aims to provide a comprehensive overview of the current understanding of SP-D’s involvement in various cancers and its potential as a biomarker and therapeutic agent, thereby offering new insights into clinical applications and future research directions.

## 2 SP-D in lung cancer

**Figure 2:**
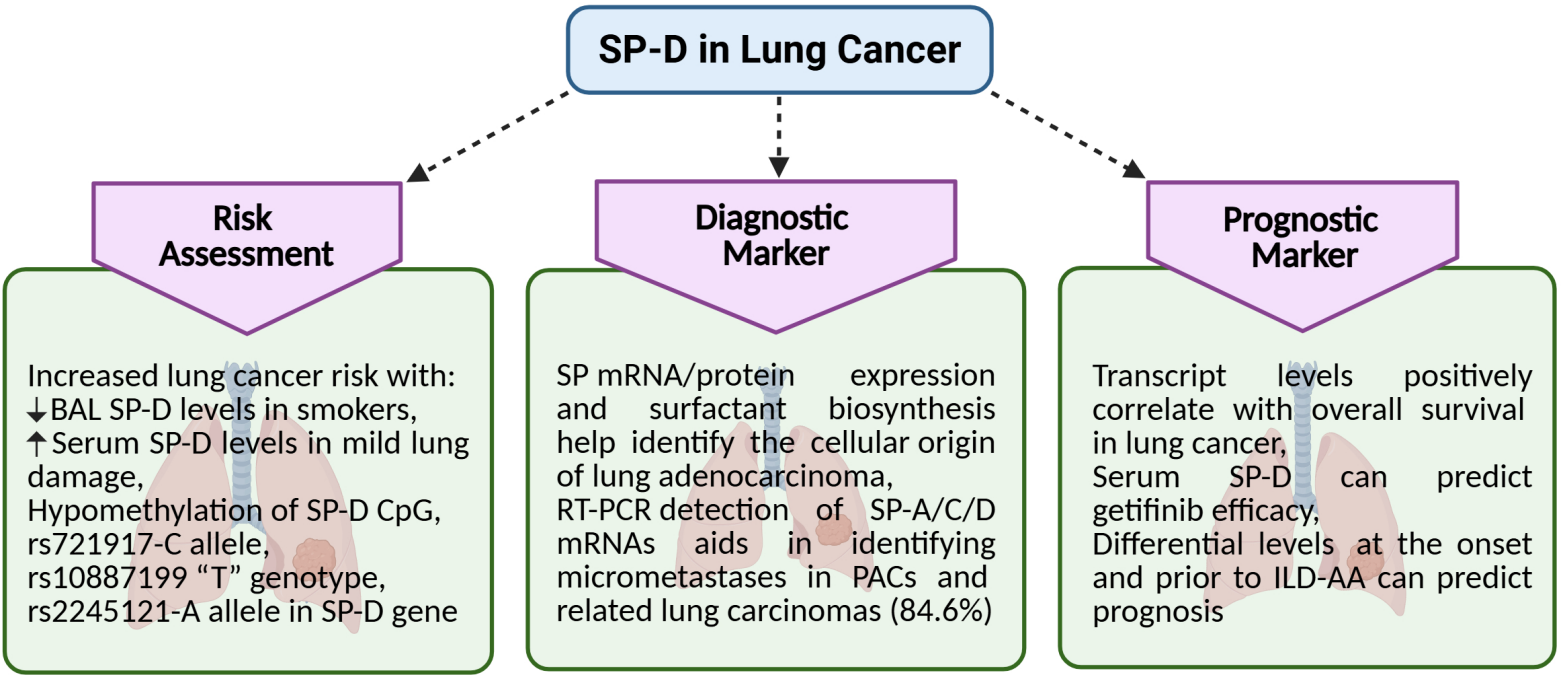
Multifaceted role of SP-D in lung cancer. SP-D exhibits diverse roles in lung cancer. It aids in risk assessment through genetic and epigenetic marker signatures. It serves as a diagnostic marker since differential expression of SP mRNAs and protein, along with estimation of surfactant biosynthesis in occult cancer cells, is useful in determining the cellular origin of lung adenocarcinoma. Detection of *SFTPA*, *SFTPC*, and *SFTPD* transcripts together by RT-PCR is useful in the detection of micrometastases of pulmonary adenocarcinomas (PACs), large cell carcinomas, and adenosquamous pulmonary carcinomas. SP-D also functions as a prognostic marker by correlating with survival, treatment response, and disease progression. SP-D mRNA expression is positively associated with overall survival in lung cancer patients, with significant prognostic value in adenocarcinoma. Patients with EGFR mutations exhibit significantly higher pre-treatment serum SP-D levels, suggesting its potential as a simple, non-invasive biomarker for predicting Gefitinib response. *Image created in Biorender.* https://BioRender.com.

Lung cancer is the most frequently diagnosed cancer, responsible for around 2.5 million new cases and the leading cause of cancer mortality (estimated 1.8 million deaths in 2022) (29). Since SP-D was originally identified to be a crucial immunomodulatory protein in the lung surfactant, its role in lung cancer was explored first. The following studies demonstrate its mechanism of action in lung cancer cells and its significance in risk assessment, diagnosis and prognosis of lung cancer (**Figure 2**).

### 2.1 SP-D inhibits epidermal growth factor receptor (EGFR) signaling in lung cancer cells

SP-D inhibited proliferation, migration and invasion of lung cancer cell lines, A549 and H441, by directly binding to EGFR via its N-glycans (30). Binding and labeling studies suggested that SP-D bound to EGFR via its CRD (30). SP-D interfered with epidermal growth factor (EGF) binding and subsequently suppressed EGF signaling. EGFR being one of the most important targets in cancer therapy, SP-D could be explored as an adjunct therapy for lung cancer especially for patients with/without *EGFR* gene mutations and those who are resistant to EGFR–Tyrosine kinase inhibitor treatment. Like SP-D, SP-A interacted with EGFR and inhibited its signaling in A549 lung cancer cells. However, SP-A binding to EGFR is not via its CRD and rather depends on its oligomerization into the dodecameric form, suggesting a distinct mechanism of action for SP-A and SP-D in inhibiting EGFR signaling (31).

### 2.2 SP-D in the assessment of risk for lung cancer

Decreased SP-D levels in bronchoalveolar lavage (BAL) fluid are associated with progression of bronchial dysplasia in heavy smokers who are at high risk of lung cancer (32). For every 1-unit decrease in the log-normalized SP-D levels, the odds of disease progression increased by 3.2-fold. Moreover, a 1-unit decrease in BAL SP-D levels over 6 months further increased the risk of disease progression by 76% (OR, 1.76; p = 0.023). Reduced BAL SP-D levels may contribute to excessive inflammation and oxidative stress in the lung promoting the formation of dysplastic lesions. BAL SP-D levels can thus serve as a novel biomarker to identify smokers who are at high risk of progression to early lung cancer.

Altered gene methylation patterns are a hallmark of cancers, including lung cancer (33). Four SP CpG markers (SP-A1_1080, SPA1_370, SP-D_1170, and SP-D_1370) are significantly hypomethylated in lung adenocarcinoma and squamous cell carcinoma making them potential biomarkers for the diagnosis of lung cancer (34). Normal lung tissues with a higher level of unmethylated SP-A1_1468 and SPD_1170 CpG exhibited higher *SFTPA1* and *SFTPD* mRNA levels, indicating that CpG methylation may play an important role in gene expression. Since SP-D (along with SP-A) are known to play crucial roles in host defense and regulation of inflammatory responses in the lung, SP gene hypomethylation resulting in altered levels of surfactant proteins may compromise proper activation of anti-tumor immune responses (35, 36).

*SFTPD* SNP rs721917 was found to be associated with lung cancer with allele “C” being the risk allele for the development of lung cancer (37). The (T→C) results in an amino acid change (Met11Thr) that causes a decreased serum concentration of SP-D with lower oligomerization and reduced binding to bacteria (38, 39). Haplotype analysis revealed that lung cancer is associated with both “T/T” and “C/T” haplotypes of rs721917 and rs10887199, indicating that the rs10887199 “T” genotype may also have an effect on lung cancer rather than the rs721917 genotype. *SFTPD* haplotypes may result in changes in the CRD, affecting the binding of SP-D to Deleted in malignant brain tumors 1 (DMBT1), a tumor suppressor thus reducing its tumor-suppressing capability. The “C” allele is also the risk allele for emphysema but not for interstitial pneumonia (IP), both conditions characterized by chronic inflammation from which lung cancer often arises. This suggests a similar mechanism of involvement of SP-D in emphysema and lung cancer but not for IP. Additionally, no significant mediating effect of emphysema or IP in the development of lung cancer has been revealed.

Likewise, rs2245121-A allele in *SFTPD* gene was correlated with an increased risk of lung cancer only in smokers (p<0.05), plausibly due to the protective role of SP-D in the lungs against inhaled harmful substances like tobacco smoke (40). An interaction between *SFTPD* gene polymorphisms and smoking may be involved in the development of lung cancer. *SFTPD* single nucleotide polymorphism (SNP) screening may thus aid in early screening for lung cancer in smokers.

Serum SP-D levels are elevated in mild pulmonary disease or damage in the absence of clinical illness and could be used along with other known predictors to identify individuals at a high risk of developing lung cancer (41). A positive association is observed between circulating levels of SP-D and subsequent risk of developing lung adenocarcinoma (OR = 1.65; p-trend=0.008) and squamous cell carcinoma (OR = 4.22; p-trend=0.0002). The possible cause for the increase in SP-D levels is attributed to proliferation of type II alveolar cells that replace type I alveolar cells due to lung injury (42) which eventually transform into type I cells or undergo EMT (43). However, a large number of type II alveolar cells persist in the presence of interstitial fibrosis and secrete high levels of SP-D (42). Elevation in SP-D level is also positively correlated with C-reactive protein (CRP), indicating that SP-D concentrations increase following lung inflammation. Chronic pulmonary inflammation could initiate or promote the development of lung cancer, causing direct genotoxic injury as a result of highly oxidative microenvironment, alteration of gene methylation, and/or increased cellular proliferation during tissue repair (44, 45) Moreover, a significant association between serum levels of SP-D and risk of developing lung cancer was observed in a prospective cohort as early as two years, indicating that lung tissue damage due to inflammation over an extended period contributes to the development of lung cancer (41).

### 2.3 SP-D in diagnosis of lung cancer

Primary lung adenocarcinomas could originate from Clara cells and alveolar type II cells, peripheral airway cells, which are the progenitor cells of the bronchioles and alveoli, respectively. Alveolar type II cells secrete surfactant that contains phosphatidylcholine (PC) and phosphatidylglycerol (PG) and the surfactant proteins-SP-A, SP-B, SP-C, and SP-D. Clara cells do not produce phospholipids but express SP-A, SP-B, and SP-D (not SP-C). Thus, the estimation of surfactant biosynthesis, detection of SP proteins and SP mRNA by real-time qPCR in occult cancer cells isolated from pleural effusions could be useful in determining the cellular origin of lung adenocarcinoma (46).

Immunohistochemical analysis has revealed the presence of occult lymph node micrometastases in about two-third of the patients whose nodes did not contain metastases following routine histological examination (47). Although RT-PCR of SP-A, SP-C, or SP-D as tumor markers is a valuable tool to detect micrometastases of pulmonary adenocarcinomas (PACs), large cell carcinomas, and adenosquamous pulmonary carcinomas, the detection rate is limited as each of these cell markers is expressed only in a subset of tumors (16). The detection rate of metastatic PACs increases up to 84.6% when SP-A, SP-C, and SP-D are evaluated together. A combination of targets (48) can increase the sensitivity of detection using RT-PCR approach.

### 2.4 SP-D in prognosis of lung cancer

As compared to normal lung, *SFTPD* mRNA level is significantly lower in lung adenocarcinoma (-7.847-fold, -7.380-fold), squamous cell carcinoma (-27.565-fold, -21.161-fold), large cell carcinoma (-58.715-fold), small cell carcinoma (-7.602-fold), and tumor carcinoid (-29.978-fold) (21). *SFTPD* mRNA expression was positively related to an overall survival rate of lung cancer patients, stratified into lung adenocarcinoma (hazard ratio/HR= 0.59{0.46-0.76}) and squamous cell carcinoma (HR = 0.74{0.58-0.95}) which was further corroborated by bioinformatics studies. A more favorable prognosis was observed for adenocarcinoma with respect to squamous cell carcinoma possibly because adenocarcinoma originates from progenitor cells that produce SP-D. Moreover, SP-D in the lung is under the control of thyroid transcription factor 1 (TTF-1). Interestingly, a recent meta-analysis has highlighted that TTF-1 overexpression is related to a favorable prognosis for non-small cell lung carcinoma patients (49). Additionally, SP-D expression was found to be lower in the TME when compared to healthy pulmonary parenchyma, suggesting an immunomodulatory role for SP-D in the lung TME (21).

Serum SP-D level can serve as a prognostic marker to predict the efficacy of gefitinib in patients with non-small cell lung cancer (NSCLC) (50). Gefitinib, an EGFR tyrosine kinase inhibitor (EGFR-TKI) is shown to be more effective in NSCLC patients with *EGFR* mutations than in patients without these mutations. Patients with *EGFR* mutations have significantly higher serum SP-D levels [Median 76.1ng/ml (56.4, 92.0) vs 41.3ng/ml (19.0, 60.9)] before treatment which can be used as a simple, non-invasive and convenient marker to predict Gefitinib efficacy rather than checking for these mutations.

Anticancer agent-induced interstitial lung disease (D-ILD) is one of the most common adverse effects of cancer treatment. Hence, the development of interstitial lung disease-anticancer agent induced (ILD-AA) can be critical for a patient’s prognosis. Amongst other serum markers such as Krebs von den Lungen-6 (KL-6), ΔSP-D (SP-D levels at the onset of ILD-AA minus the values prior to ILD-AA) was found to be the most promising biomarker to predict the prognosis of ILD-AA (51). Serum SP-D levels were significantly higher at the onset of ILD-AA in patients who died of ILD-AA as compared to the survivor group. Moreover, ΔSP-D in the death group was significantly higher than in the survival group (284 ± 114.30 ng/mL vs −126 ± 67.91 ng/mL, p = 0.033). Although SP-D levels were also found to increase in a few patients in the survival group, the change was significantly lower when compared to patients in the death group. Interestingly, patients with low ΔSP-D also had significantly longer survival times than those with high ΔSP-D (Median survival time/MST of 159 days; 95% confidence interval/CI, 72–328 vs MST of 30 days; 95% CI, 3–33 in high ΔSP-D, Hazard ratio/HR: 26.02, p = 0.001, by log-rank test). Of the six variables analyzed (age, smoking history, performance status, presence of emphysema, presence of interstitial shadow, high ΔSP-D), only high ΔSP-D level was found to be a significant independent risk factor for mortality in patients with ILD-AA, consistent with High-Resolution Computed Tomography (HRCT) findings.

## 3 SP-D in other cancers

Although initially believed to be only a part of the lung surfactant, SP-D expression has been reported in many extra-pulmonary tissues such as brain, trachea, salivary gland, heart, mammary gland, stomach, small intestine, pancreas, kidney, prostate gland, uterus, testis and placenta (4), suggesting immunomodulatory roles at these sites. Several studies have reported the prognostic role of SP-D in various cancers (**Table 1**).

**Table 1:** Expression profile of Surfactant Protein D in pulmonary and various non-pulmonary cancers and associated clinical prognostic significance.

Sr. no.	Cancer type	SP-D expression profile	Clinical significance
1.	Lung Cancer	Compared to normal lung tissue, lower SP-D mRNA expression in: - lung adenocarcinoma, - squamous cell carcinoma, - large cell carcinoma, - small cell carcinoma, - tumor carcinoid (21)	Positive correlation between SP-D expression and an overall survival rate of the patients with lung cancer (21)
2.	Ovarian Cancer	Higher transcript levels of SP-D in serous cystadenocarcinoma than normal ovary (21)	Negative association between SP-D levels and overall/ progression-free survival rates of patients with stage-1 and -2 serous cystadenocarcinoma, No correlation observed for patients with stage-3 and -4 ovarian cancer (21)
3.	Breast Cancer	Compared to normal breast tissues lower SP-D mRNA expression in: - invasive ductal breast carcinoma tissues, - male breast carcinoma tissues (21)	Negative association between SP-D levels and a favorable prognosis in breast cancer patients with Luminal-A only grade-1 and -2 cancers, No correlation observed for patients with Luminal-B, HER2+, Basal, grade-3, mutated p53, wild-type p53 cancers (21)
4.	Gastric Cancer	Higher SP-D transcript expression in healthy gastric mucosa compared to malignant tissues stratified into intestinal, diffuse, and mixed-type adenocarcinoma (21)	A higher expression of SP-D was negatively related to an overall survival rate in the gastric cancer patients, This negative prognostic association was significant in patients with intestinal-type adenocarcinoma, those without distant metastasis, and HER2-negative cases (21)
5.	Prostate Cancer	Decreased SP-D protein expression in prostate adenocarcinoma compared to non-malignant prostate tissue (17)	Decrease in SP-D levels with increasing Gleason score and tumor volume indicate that it might be a negative prognostic factor in prostate adenocarcinoma (17)

### 3.1 Breast cancer

SP-D is expressed in mammary glands (4) and has been implicated in modulating immune responses in breast tissue (21). Interestingly, in contrast to its role in lung cancer, higher SP-D expression is linked to poorer survival rates in breast cancer patients. This suggests a tissue-specific function of SP-D, particularly in modulating immune responses or inflammation within the TME.

A crucial interaction between SP-D and extracellular matrix (ECM) in promoting the progression and evasion of breast cancer was reported (52). In their *in vitro* study, treatment with rfhSP-D reduced the viability of Human EGFR2 (HER2) overexpressing SKBR3, triple-positive BT474, and MCF-7 breast cancer cells. In SKBR3 and BT474 cell lines, rfhSP-D induced G2/M cell cycle arrest and activation of the intrinsic apoptotic pathways. Hyaluronic acid (HA), an important constituent of ECM, plays a central role in modulating the inflammation and cellular migration in cancer and the associated tumor progression. HA negated the apoptotic effects of rfhSP-D on breast cancer cells by diminishing the transcriptional levels of p53 in rfhSP-D-treated SKBR3 cells (52). These findings underscore the complex interactions between SP-D and the ECM components.

### 3.2 Gastrointestinal cancers

Unlike lung cancer, low SP-D levels are associated with a favorable prognosis in gastric cancers (21). In gastric cancers, low CD11c expression is correlated with poor prognosis (53), suggesting that SP-D mediated suppression of CD11c can impair immune surveillance within the TME, thereby contributing to tumor progression (25). This modulatory effect of SP-D on immune cells is interesting as lower SP-D expression leads to worse clinical outcomes in gastrointestinal cancers (21).

### 3.3 Pancreatic cancer

The involvement of SP-D in pancreatic cancer is complex, and its native expression within the TME and serum requires clinical validation. Experimental studies demonstrate that SP-D actively suppresses pancreatic cancer cell progression by inducing apoptosis and suppressing EMT (20). Treatment with rfhSP-D induces G1 cell cycle arrest and the transcriptional upregulation of pro-apoptotic factors, such as tumor necrosis factor-alpha (TNF-α), nuclear factor kappa B (NF-κB), and the pro-apoptotic marker Fas, in human pancreatic adenocarcinoma cell lines, including Panc-1 (p53^mt^), MiaPaCa-2 (p53^mt^), and Capan-2 (p53^wt^). This suggests that rfhSP-D can potentially be used to target pancreatic cancer cells irrespective of their p53 phenotype (19). The inflammatory TME in pancreatic ductal adenocarcinoma (PDAC) is an important aspect that contributes to EMT, invasion, and tumor metastasis. Treatment with rfhSP-D may suppress the invasive-mesenchymal properties of highly aggressive pancreatic cancer cells by inhibiting Transforming Growth Factor Beta (TGF-β) and Smad2/3 expression, thereby limiting their invasive potential (20). This was further confirmed by reduced levels of key EMT genes such as Vimentin (*VIM*), Zinc-finger E-box binding homeobox 1 (*ZEB1*), and Snail family transcriptional repressor 1 (*SNAI1*) upon rfhSP-D treatment in these cells (20).

### 3.4 Ovarian cancers

The presence of SP-D has been reported in ovarian cancer, with high transcriptional levels and detection in circulating tumor cells of ovarian cancer patients, suggesting its potential suitability as a biomarker (54). Oncomine analysis, coupled with immunohistochemical scoring, revealed widespread *SFTPD* mRNA expression in ovarian cancer tissues across different cancer stages (54). *In vitro* studies using rfhSP-D demonstrated its pro-apoptotic effects in SKOV3, a human ovarian clear cell adenocarcinoma cell line that lacks functional TP53 expression (54), further highlighting the involvement of a p53-independent mechanism. However, in the *in vivo* TME, SP-D’s protective effects appear to be compromised, demonstrating an unfavorable prognosis (21). The significant correlation of unfavorable disease prognosis with elevated levels of SP-D in circulating tumor cells could be a survival mechanism by the tumor cells to induce suppression of immune cells attacking them. These tumor cells have likely blocked the apoptotic pathways induced by high intracellular levels of SP-D.

### 3.5 Prostate cancer

Expression of SP-D in the human prostate was noted in several studies, indicating a significantly reduced expression in advanced prostate cancers. Reduced levels of SP-D in prostate cancer tissues are associated with higher Gleason scores, increased tumor volume, and poor clinical outcomes (17), suggesting its role as a disease marker. *SFTPD* mRNA expression is markedly reduced, and SP-D protein undergoes increased proteolytic degradation in both early and late stages of prostate cancers. Thakur et al. (2019) reported lower *SFTPD* mRNA and protein in androgen-dependent prostate cancer cells (LNCaPc) compared to primary prostate epithelial cells and androgen-independent prostate cancer cell lines (PC3 and DU145) (22). *In vitro* and ex vivo studies with rfhSP-D demonstrated its ability to bind prostate cancer cells, induce apoptosis via p53 and pAkt pathways, and trigger cell cycle arrest. Mass spectrometric studies revealed GRP78 as a key interacting partner of SP-D in prostate cancer cells, potentially interfering with pro-survival signaling (23). A study by Ganguly et al. (2022) highlighted the role of ECM proteases in cleaving SP-D and altering SP-D’s anti-tumor effects using TRAMP (transgenic adenocarcinoma of mouse prostate) model (24). In particular, serine proteases secreted by granulocytes and polymorphonuclear myeloid-derived suppressor cells (PMN MDSCs) contributed to the degradation of SP-D. Administration of Sivelestat, an elastase inhibitor along with rfhSP-D, reversed these effects by promoting macrophage polarization towards M1 phenotype and downregulating PMN MDSCs in short-term, ex-vivo cultured TRAMP prostate biopsies. These findings underscore SP-D’s anti-tumorigenic role in prostate cancer while highlighting how the TME undermines its protective functions through transcriptional suppression and proteolytic degradation.

## 4 SP-D and carcinogenic pathogens

Several bacterial, viral, and fungal pathogens have been reported to contribute to cancer initiation and progression in humans, and many of these are categorized as Group I human carcinogens by the International Agency for Research on Cancer, IARC (55). They can promote cancer development by three different mechanisms: chronic infection resulting in inflammation and DNA damage, induction of oncogene expression, and host immunosuppression. Some of the pathogens linked with cancer development include *Helicobacter pylori*, Epstein-Barr virus, Hepatitis B and C virus, *Aspergillus flavus*, Human Papillomavirus, *Candida albicans* and Kaposi’s Sarcoma Herpes Virus. The section below discusses the role of SP-D in inhibiting the growth and infectivity of some of these carcinogenic pathogens (**Table 2**).

**Table 2:** Surfactant protein D interactions with carcinogenic pathogens.

Cancer type	Causative agents/risk factors	SP-D interaction and effects
Gastric cancer (56)	*Helicobacter pylori*	SP-D binds to LPS extracted from *H. pylori* and causes agglutination in a lectin-specific manner, reducing motility.
Oral cancer (66)	*Candida albicans*	SP-D binds to *C. albicans* in a calcium-dependent manner, significantly inhibiting fungal growth.
Hepatocellular carcinoma (HCC) (73)	*Aspergillus flavus*	High concentrations (60–120 μg/mL) of rfhSP-D inhibits growth of *A. flavus*.
Lung cancer (76)	Influenza virus (risk factor)	SP-D binds to Influenza A viral hemagglutinin and induces viral aggregation.

### 4.1 Helicobacter pylori

*Helicobacter pylori* (*H. pylori*) is a Gram-negative bacterium that colonizes the gastric mucosa. Once infection is established, gastric mucosal inflammation develops and may result in duodenal ulcers, gastric ulcers, gastric carcinoma and lymphoma (56). Categorized as a group I carcinogen by the IARC in 1994 (57), *H. pylori* induces chronic inflammation, which leads to increased oxidative stress, DNA damage, and impaired DNA repair mechanisms, ultimately resulting in accumulation of mutations in the gastric epithelial cells. This specifically occurs when the bacteria carry the cytotoxin-associated gene A (*CAGA*) virulence factor that interferes with signaling pathways and promotes cell proliferation, resulting in the eventual development of gastric cancer (57). SP-D is present in the gastric mucosa and its expression levels significantly increase in *H. pylori*-associated gastritis (56). SP-D can bind to LPS extracted from *H. pylori* and cause its agglutination in a lectin-specific manner resulting in a significant reduction (50%) in its motility. These observations suggest a potential role for SP-D in modulating the innate immune response to *H. pylori* infection and influencing disease progression.

### 4.2 Candida albicans

There is emerging evidence that Candidiasis may not just coexist with oral cancer, but that *C. albicans* may play a role in initiating or promoting oral cancer development (58) *Candida* exerts its carcinogenic effects on the mucosal epithelium by production of carcinogens like acetaldehyde (59, 60) and nitrosamine (61). Acetaldehyde causes accumulation of point mutations and chromosomal aberrations in cells of the oral cavity by producing DNA and protein adducts that interfere with normal DNA replication (62). Additionally, acetaldehyde affects the DNA repair enzymes, binds to glutathione and causes mitochondrial damage, thus increasing the levels of reactive oxygen species (ROS), which further adds to DNA damage (62, 63). Candida from oral cancer patients is metabolically more active and produces higher levels of acetaldehyde as compared to healthy controls (64). SP-D is reported to be expressed in the oral cavity of humans (65). *In vitro* studies have demonstrated SP-D binding to *C. albicans* in a calcium-dependent manner and is associated with significant inhibition of fungal growth (66). These observations are consistent with a potential role for SP-D in modulating oral Candida colonization and host–fungal interactions in the context of oral health.

### 4.3 Aspergillus flavus

*Aspergillus flavus* produces the hepatotoxic and hepatocarcinogenic compound, aflatoxin (67), which when ingested, may result in acute aflatoxin poisoning causing abdominal pain and vomiting. Acute exposure can cause pulmonary edema, fatty liver, liver necrosis, and even death (68). As aflatoxins are metabolized in the liver, chronic exposure to the toxin is reported to increase the risk of hepatocellular carcinoma (HCC) in humans (69). Of the four aflatoxins, AFB1 is the most common and has been strongly associated with HCC (68). Once ingested, AFB1 gets activated by microsomal enzymes in the liver, forming DNA adducts (70) that can cause G > T mutagenesis and all G > T, G > A, G > C and single nucleotide deletions in *TP53* (71, 72). High concentrations (60–120 μg/mL) of rfhSP-D is reported to inhibit growth of *A. flavus* (73) These observations suggest a possible role for rfhSP-D in influencing *A. flavus* growth dynamics and aflatoxin-related outcomes.

### 4.4 Other carcinogenic pathogens

SP-D also interacts with a range of pathogens that are known to increase the risk of developing cancer, indirectly by causing inflammation or inducing an immunosuppressive state. rfhSP-D inhibits the growth of *Aspergillus fumigatus* (73). *A fumigatus* is known to produce gliotoxin, a mycotoxin that is immunosuppressive in nature (74) and may thus contribute to the development of cancer in immuno-compromised individuals. Exposure to influenza is associated with an increased risk of lung cancer and cumulative exposure further enhances this risk (75). SP-D plays an important role in limiting Influenza A virus infectivity by binding to viral hemagglutinin and by inducing viral aggregation (76). These findings suggest that SP-D may influence host–pathogen interactions and inflammatory responses in ways that are relevant to long-term respiratory health.

## 5 Therapeutic potential of SP-D

Exogenous SP-D has shown potential as a therapeutic intervention for respiratory conditions like neonatal respiratory distress syndrome (RDS) and Bronchial dysplasia (BPD). Animal studies using Survanta, an intratracheal suspension of pulmonary surfactant made from animal lung extract, showed pronounced treatment effects in a preterm lamb model when enriched with SP-D as opposed to those receiving surfactant alone (77) *In vivo* studies using house dust mite allergen challenged mouse model (78), and bleomycin induced pulmonary fibrosis in mice (12), exogenous recombinant SP-D reduced fibrotic depositions in the lung highlighting its potential in treatment of various pulmonary diseases characterized by fibrotic deposition like allergic asthma, chronic obstructive pulmonary disease (COPD) and idiopathic pulmonary fibrosis. However, local administration may be preferred over systemic SP-D induction due to its complex role in lipid metabolism and pro-inflammatory effects in vessel walls that can enhance the risk of atherosclerosis (79). Additionally, studies suggest that SP-D has a pro-inflammatory role in the intrauterine compartments in mice during fetoplacental development in contrast with its anti-inflammatory effect in the lungs (80), highlighting the need for locally delivering SP-D or a tissue specific inhibition of SP-D.

A number of studies also suggest the potential use of modified SP-D like point substituted SP-D complexed with monoclonal antibody (81, 82) and mutant forms of recombinant human SP-D with N-linked glycosylation of CRD and porcine SP-D-specific Ser-Gly-Ala loop inserted in the CRD (83) as therapeutic options for Influenza A virus. Other prospective therapeutic applications of SP-D include use of protease-resistant SP-D in the treatment of *P. aeruginosa* (84, 85), use of SP-D in nasal spray for management of chronic rhinosinusitis (86), reduction of intestinal injury and inflammation in *Staphylococcus aureus* pneumonia (87), protection against fatal challenge with *Aspergillus fumigatus* conidia and development of pulmonary aspergillosis (88) as well as in reducing lung inflammation and injury induced by transplantation of allogeneic hematopoietic stem cells (89).

Airway Therapeutics, a biopharmaceutical company established in 2011 is focused on the development of a novel recombinant human Surfactant protein D (rhSP-D), an engineered version of endogenous human SP-D, called Zelpultide alfa (AT-100) for the treatment of BPD which affects very preterm infants. They have successfully completed a randomized blinded Phase 1b study in US and Europe in 37 infants in which no dose limiting toxicities were found and indications of efficacy were observed, and have received approval for Phase 3 trial (90). Another clinical trial, called ‘Recombinant surfactant protein D to prevent neonatal chronic lung disease-Safety trial (RESPONSE)’, is a first-in-human study of rfhSP-D and aims to find out the safest preventive dose of rfhSP-D for premature infants (under 30 weeks gestation) who are at high risk of chronic lung disease (CLD) (91). Although there are several patents filed for SP-D, most of them focus on the use of SP-D as a biomarker for cancer and various diseased conditions. To take the therapeutic development of SP-D and rfhSP-D forward in cancer research, a structured translational roadmap is essential. The next phase should focus on systematically building therapeutic evidence to take SP-D from bench to bedside by elucidating its molecular targets and signaling pathways related to apoptosis, immune modulation and TME remodeling using transcriptomic and proteomic analyses. This should be complemented with the development of scalable recombinant production systems and rigorous validation of its therapeutic efficiency using xenograft animal models. The clinical application of SP-D as an anti-cancer therapeutic is hindered by several challenges. Despite multiple studies demonstrating tumor-suppressive functions of SP-D, its role in cancer remains context-dependent, influenced by tumor type and the TME, thus complicating the prediction of therapeutic responsiveness. Further, the specific molecular mechanisms of SP-D-mediated anti-tumor activity have not been fully elucidated using appropriate *in vivo* mouse tumor models. Since SP-D is a large multimeric glycoprotein, for its therapeutic application, evaluation of factors such as its route of administration, effective biodistribution, half-life, proteolytic degradation and immunogenicity is important. Given the multifaced roles of SP-D and its ability to interact with many different cell types, it is also important to assess the off-target effects. Finally, the combination of SP-D into current oncology treatment paradigms requires a deeper understanding of its interactions with chemotherapy, radiotherapy, targeted agents and immunotherapies for better cancer management.

## 6 Discussion and conclusions

**Figure 3:**
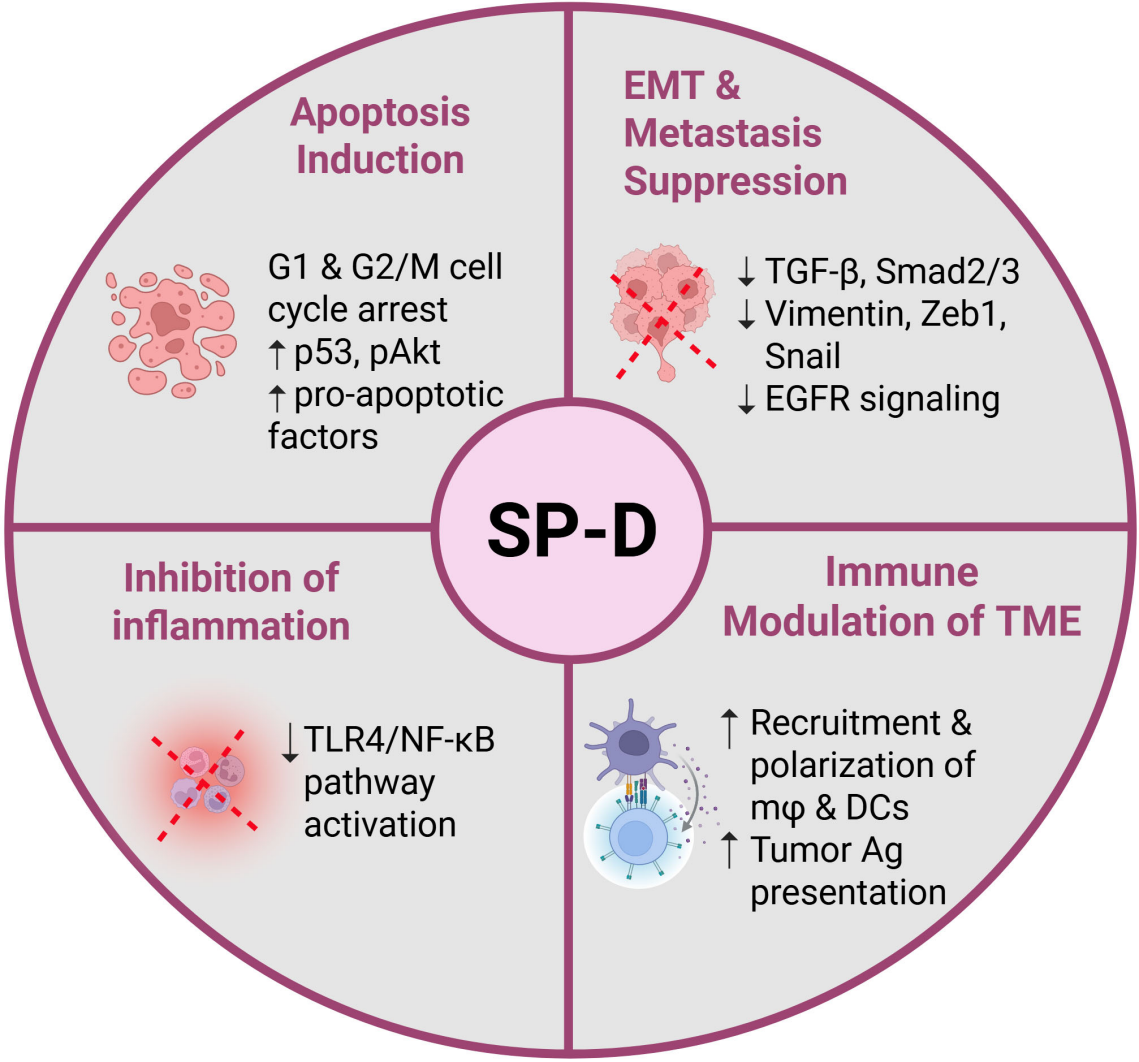
Anti-tumoral effects of SP-D. SP-D demonstrates anti-tumoral effects on cancer cells by, (1) Apoptosis induction: rfhSP-D induces G1 and G2/M cell cycle arrest and apoptosis *via* p53 and intrinsic pathways in eosinophilic leukemia, breast, prostate, and pancreatic cancer cells. (2) Suppression of EMT and metastasis: rfhSP-D downregulates TGF-β and Smad2/3 signaling and mesenchymal markers such as vimentin, Zeb1, and Snail in pancreatic cancer cells. It also inhibits EGFR-mediated proliferation and invasion in lung cancer cells. (3) Inhibition of TLR-mediated tumor promotion: TLR4/NF-κB signaling is implicated in inflammation-driven tumorigenesis in various cancer types including esophageal squamous cell carcinoma, colon and liver cancers. Since SP-D is reported to interfere with TLR4/NF-κB signaling, it can inhibit inflammation mediated tumor progression. (4) Modulation of the tumor microenvironment (TME): SP-D can influence recruitment and polarization of immune cells such as macrophages (mΦ) and dendritic cells (DC) in TME, and may enhance DC trafficking, maturation, and tumor antigen presentation to support anti-tumor immunity. *Image created in Biorender*. https://BioRender.com.

Altered SP-D levels in BAL, serum and tissue biopsies can serve as prognostic markers in lung and non-lung cancers. *In vitro* studies suggest anti-tumorigenic potential of SP-D against different types of cancer; however, its role *in vivo* is greatly influenced by the TME. While SP-D presence correlates with better lung cancer outcomes, it fails to protect against other cancers, likely because of tissue-specific variations in the TME. Increased HA in the breast TME acts as a shield, deterring the ability of SP-D to induce apoptosis in cancer cells (52). Targeting HA synthesis and accumulation in the breast TME presents a promising therapeutic strategy that can remove the HA-driven suppression of the pro-apoptotic activity of rfhSP-D. Over-secretion of serine proteases and elastases in the prostate TME promotes the proteolytic cleavage and inactivation of SP-D, thereby inhibiting its tumor-suppressive functions in prostate cancer (24). The poor prognosis in gastric cancer linked to SP-D may be due to SP-D-mediated suppression of CD11c (25), an activation marker on antigen-presenting cells. This in turn may cause immune quiescence and inhibition of anti-tumor responses within the TME. Like gastric cancer, elevated SP-D levels may cause an immunosuppressive state in the TME of ovarian cancer, thus resulting in poor clinical outcomes (21). While ELISA is widely used for SP-D detection, it lacks specificity in distinguishing functional SP-D from its dysfunctional fragments, making western blot a more reliable assay for assessing structurally intact biologically active SP-D (85). Hence, assay selection should be carefully considered for assessing SP-D expression levels and there is a need to develop point-of-care tests that enable distinctive detection of functional and dysfunctional forms of SP-D. Beyond its prognostic value, SP-D exhibits direct anti-cancer properties, including G1 and G2/M cell cycle arrest, apoptosis induction, EMT inhibition and inhibition of EGFR signaling. Inflammation driven tumor progression is a hallmark of various malignancies, including esophageal squamous cell carcinoma, colorectal cancer, gastric cancer and HCC (92). Through its ability to inhibit the activation of TLR4/NF-κB pathway, SP-D serves as a key regulator of inflammation (10, 93) and may thereby exert indirect anti-tumorigenic effects. Additionally, SP-D plays a critical role in the regulation of immune responses by modulating the recruitment and polarization of innate immune cells, particularly macrophages and dendritic cells (DC) (10). By promoting DC maturation and trafficking (94), SP-D can potentially facilitate tumor antigen presentation, aiding the adaptive immune response against cancer cells (**Figure 3**). Full-length SP-D and rfhSP-D, hence, emerge as promising therapeutic candidates for cancer, warranting further exploration for pre-clinical and clinical application.
